# Supplementary material for: Non-associative phase separation in an evaporating droplet as a model for prebiotic compartmentalization
Source: Nat Commun. 2021 May 27;12:3194. doi: 10.1038/s41467-021-23410-7 (PMC8160217; doi:10.1038/s41467-021-23410-7)
Supplement: Supplementary file 1 — Supplementary information [file 41467_2021_23410_MOESM1_ESM.pdf]

Supplementary Information

Non-associative phase separation in an evaporating droplet as a model for prebiotic compartmentalization

Wei Guo,[†,⁋] Andrew Kinghorn,[‡,⁋] Yage Zhang,[†] Qingchuan Li,[†] Aditi Dey Poonam,[†] Julian A. Tanner,[‡,§,*]
Ho Cheung Shum[†,§,*]

† Department of Mechanical Engineering, Faculty of Engineering, The University of Hong Kong, Hong Kong (SAR), China

‡ School of Biomedical Sciences, LKS Faculty of Medicine, The University of Hong Kong, Hong Kong (SAR), China

§ Advanced Biomedical Instrumentation Centre, Hong Kong Science Park, Shatin, New Territories, Hong Kong (SAR), China

⁋ These authors contributed equally to this work.

* To whom correspondence should be addressed:

Email: jatanner@hku.hk

ashum@hku.hk

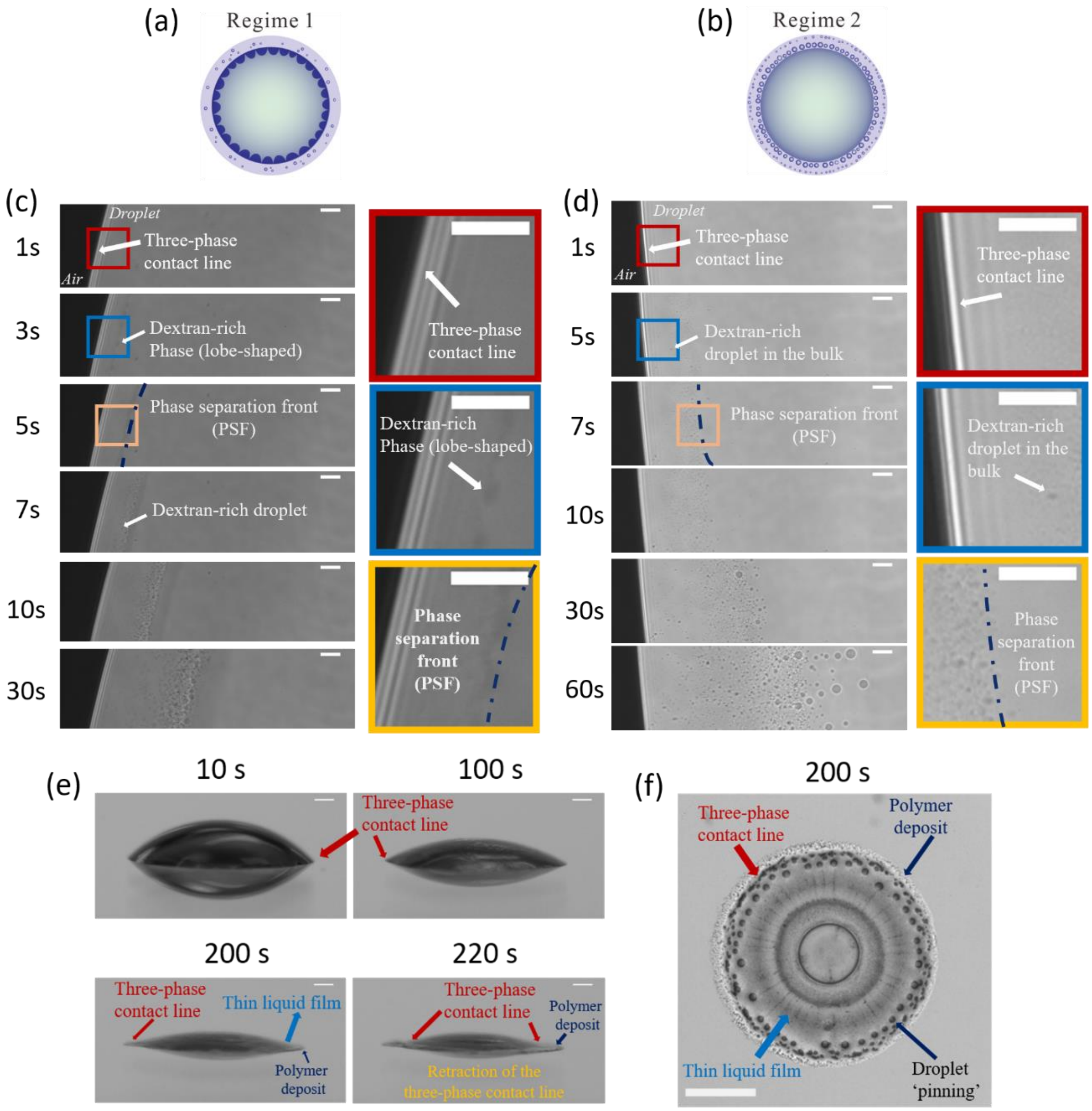

Supplementary Figure 1. Nucleation of dextran-rich domains at the three-phase contact line. (a-b) Schematics of phase-separated patterns in regime 1 and regime 2. Single-phase region (light green color) is distinct from the phase separated region (light purple for PEG-rich phase and dark purple for dextran-rich phase) inside the sessile droplet. (c) Image sequences of dextran-rich phase nucleation at the contact line in regime 1 (Scale bar: 20 μm). (d) Image sequences of dextran-rich phase nucleation at the contact line in regime 2 (Scale bar: 20 μm). The dynamics near the three-phase contact line of both regime 1 and regime 2 are discussed in Supplementary Note 1. (e) and (f) show the reduced droplet height due to water evaporation (Scale bar: 100 μm) and the 'pinning' of dextran-rich droplets near the three-phase contact line (Scale bar: 500 μm), respectively. We attribute the 'pining' effect to the reduced droplet height that inhibits Marangoni convection. Specifically, at the early stage, the sessile droplet (0.5 μL) has a relatively large height of about 200 μm, which leaves sufficient space for the development of the inward Marangoni flow. As a result, most of the nucleated droplets near the three-phase contact line can be

convected to droplet center. Further evaporation will cause the decrease of droplet height, until only a thin liquid film is left near the three-phase contact line. At this time point, it is difficult for dextran-rich droplets near the rim to move further. These droplets stop moving inward but only keep growing as they coalesce with the nucleated droplets from phase separation of the liquid film, as shown in Supplementary Fig. 1(f). Images are obtained over analysis of seven independent trials with relative humidity ranging from 55% to 65%.

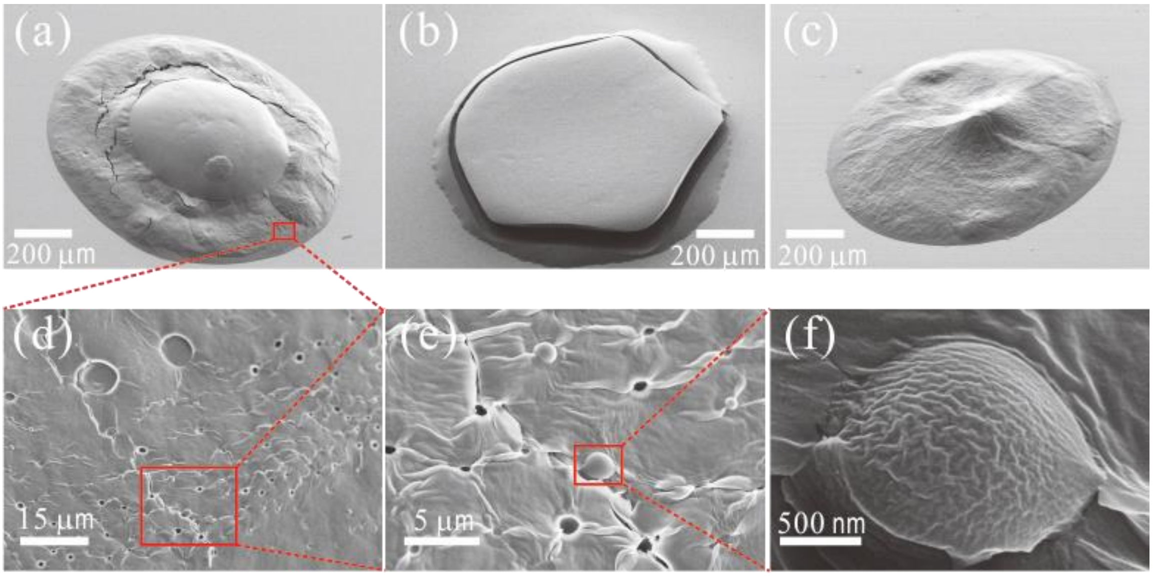

Supplementary Figure 2. SEM images of the droplet deposit left after evaporation. (a), (b) and (c) show the deposition of a sessile droplet composed of 5wt% PEG and 10wt% dextran mixture, 10 wt% dextran solution, as well as the 10 wt% PEG solution, respectively. We can see that the surface morphology of PEG solution ((c)) droplet deposited has much more stripes than that of dextran droplet deposited ((b)), which looks much smoother. (d-f) Nano-scaled compartments in the deposited droplets. Images are obtained over analysis of at least three independent trials with relative humidity ranging from 50% to 75%.

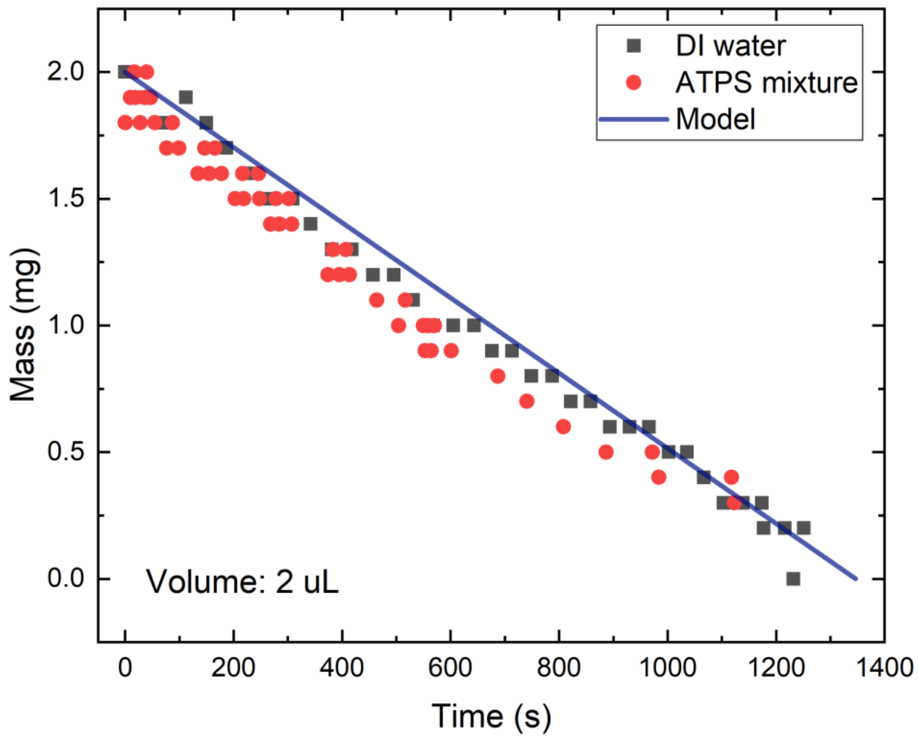

Supplementary Figure 3. Measured droplet mass as a function of evaporation time. A droplet with a volume of 2 µL was pipetted onto the glass. The mass of the droplet was recorded by an analytical balance. Raw data of an individual measurement is presented without giving mean and error values. Both droplets of DI water and ATPS mixture show a linearly decreasing mass during evaporation. The solid line is obtained by calculating evaporating rate based on Equation 1 in the main text. Source data are provided as a Source Data file.

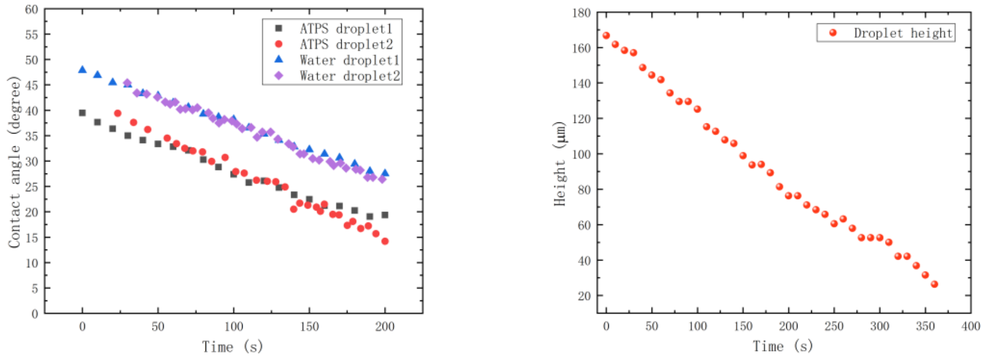

Supplementary Figure 4. Geometrical parameters of droplet during evaporation. (a) The decreasing contact angle of the sessile droplet during evaporation. Raw data of two individual measurements are presented without giving mean and error values. The change of contact angle of the ATPS droplet is consistent with that of pure water droplet, except for their initial values. (b) Droplet height of an ATPS droplet as a function of evaporation time. Raw data of an individual measurement is presented without giving mean and error values. Source data are provided as a Source Data file.

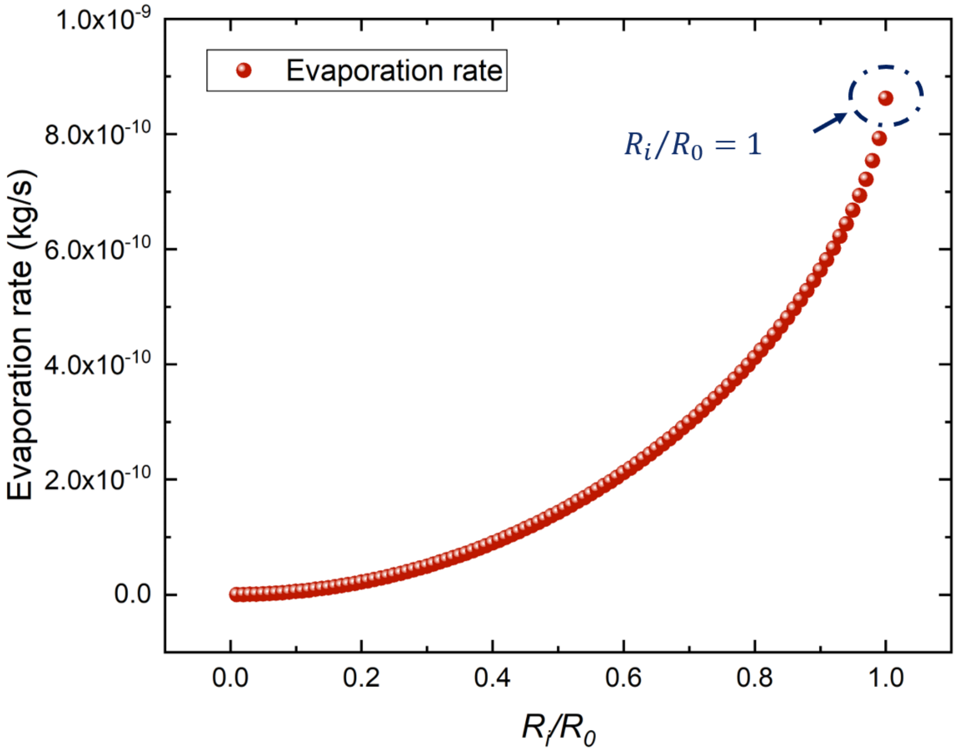

Supplementary Figure 5. Evaporation rate from the center of the sessile droplet ($R_i = 0$) to the edge of the sessile droplet ($R_i = R_0$). The non-uniform evaporation flux along the droplet surface determines the local evaporation rate. Note that the last data point denotes the total evaporation rate of the sessile droplet, about 0.90 μg s^{-1}. This value is quite close to the experimentally measured evaporation rate, which is 1.06 ($\pm$ 0.03) μg s^{-1}. Source data are provided as a Source Data file.

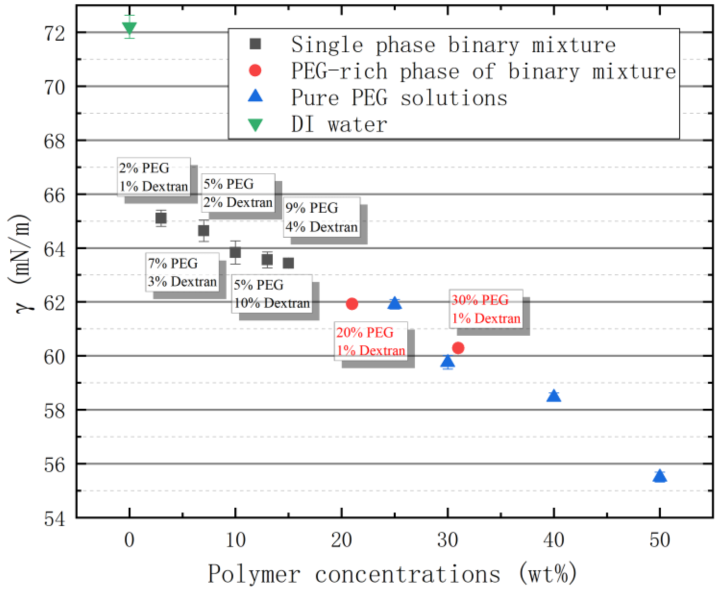

Supplementary Figure 6. A plot of the measured surface tension of the air-liquid interfaces as a function of polymer concentrations. The liquids tested include pure water, single-phase ATPS mixture, PEG solution, as well as phase-separated PEG-rich component. The surface tension was measured using the pendant droplet method[1 2], with a custom MATLAB code. Error bars represent S.E.M. (standard error of the mean) from five independent experiments. Source data are provided as a Source Data file.

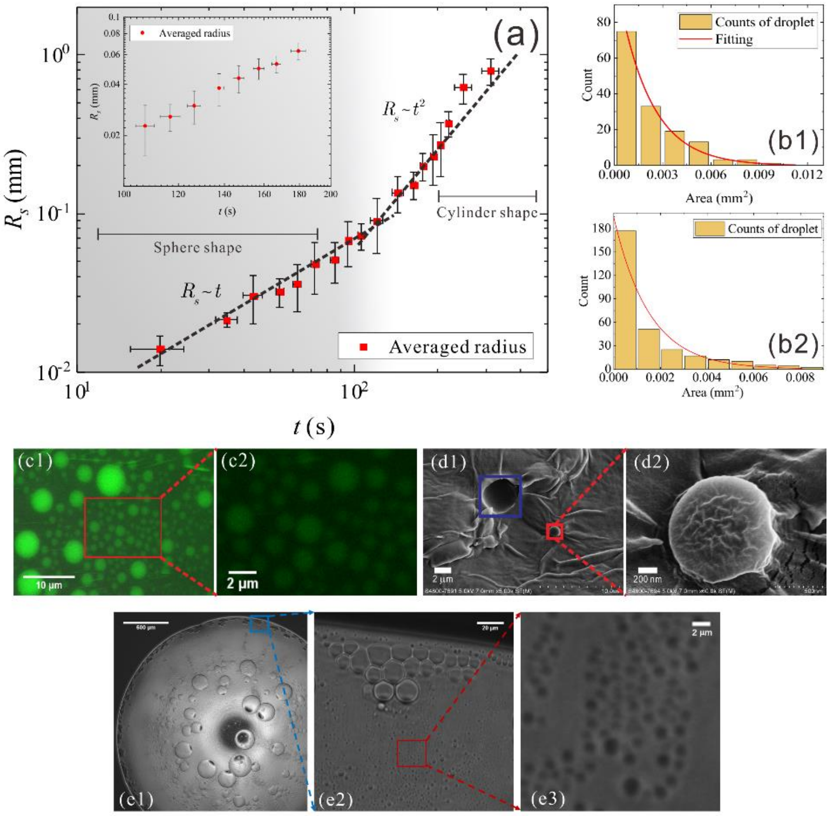

Supplementary Figure 7. (a) Time evolution of the average radius of dextran-rich droplets formed by LLPS in regime 2; Insert: Average radius evolution of PEG-rich droplets dispersed in the dextran-rich compartments in regime 1. The analysis of the scaling is provided in Supplementary Note 2. Error bars represent S.E.M. from three independent experiments. (b) Size distribution of the phase-separated droplets in regime 1 (b1) and regime 2 (b2). The size is measured at a well-defined time point. Specifically, for the droplet in regime 1, we measure the size distribution of phase-separated PEG-rich droplets inside the dextran-rich region, just before they finally coalesce together. For the droplet in regime 2, we measure the size distribution of phase-separated dextran-rich droplets just before the phase separation front (PSF) moves to the center of the sessile droplet. The two size distributions of these small droplets both follow an exponential decay distribution. (c1, c2) Confocal microscope images of nucleated dextran-rich compartments inside the sessile droplet; (d1, d2) SEM images showing dehydrated nano-scale compartments in the deposited sessile droplet; (e1, e2, e3) Phase contrast images showing submicron-sized dextran-rich droplets near the rim, with a scale bar of 500 μm, 20 μm and 2 μm, respectively. For (c) and (e), images are obtained over analysis of seven independent trials with relative humidity ranging from 55% to 65%. For (d), images are obtained over analysis of three independent trials with relative humidity ranging from 50% to 75%. In our model system, if it is assumed that there are no more than 10 polymer chains inside the phase-separated “nuclei” in PEG/dextran (the phase-separated “nuclei” of the ternary mixture of polyethyl-butylene, polymethylbutylene, and apolymethylbutylene-block-polyethylbutylene copolymer contains only a few polymer chains and has a size ranging

from 20 nm to 50 nm.[3]), the size of the nuclei could also be in the range of a few tens of nanometers, given that the polymer gyration radius of PEG and dextran is a few nanometers. Source data are provided as a Source Data file.

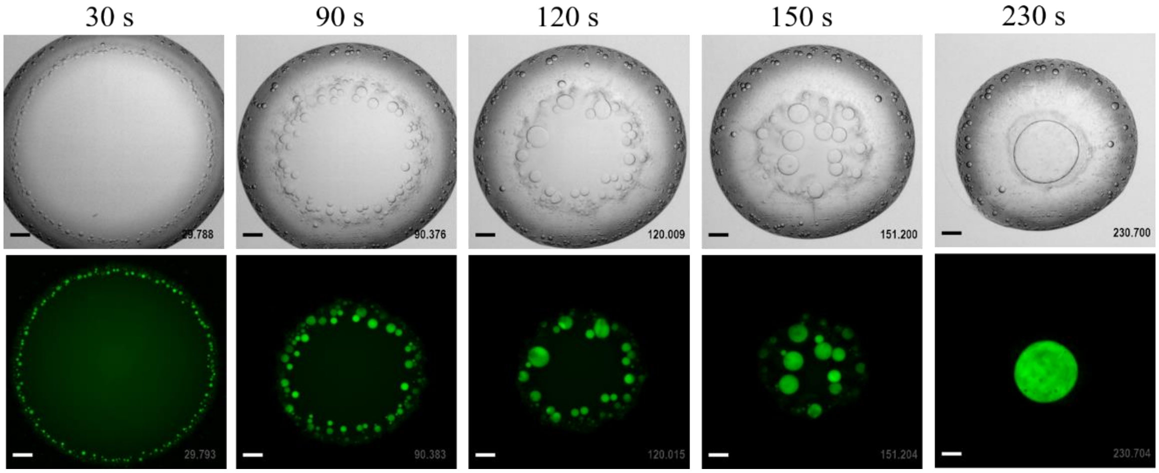

Supplementary Figure 8. Localization of fluorogenic RNA aptamer inside the evaporating sessile droplet. Sequences of fluorescence images (upper panel) and of bright field images (lower panel) suggest that RNA is localized into dextran-rich compartments during the LLPS process. Images are obtained over analysis of seven independent trials with relative humidity ranging from 55% to 65%. The scale bar is 200 μm.

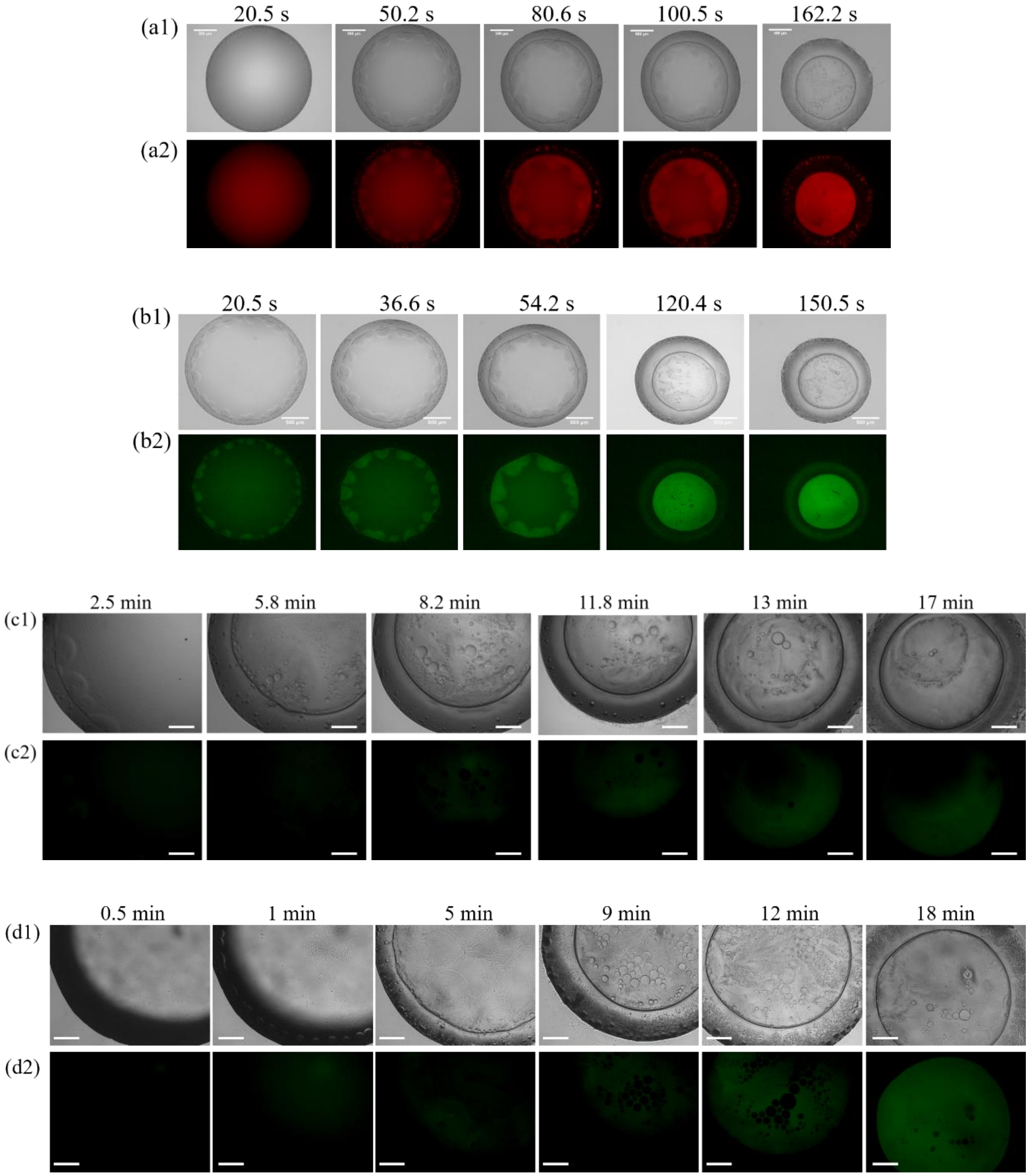

Supplementary Figure 9. Image sequence of DNA partitioning (a), RNA partitioning (b), DNA transcription (c) and ribozyme cleavage (d) inside the evaporating ATPS droplet of regime 1. The upper panel and lower panel represent the bright-field and fluorescence image sequence, respectively. The ATPS mixture is composed of 5 wt% PEG and 10 wt% dextran. In (a) and (b), a droplet of 0.5 µL was pipetted onto the glass slide, with 10 µM CY-5 labeled DNA (length of 25 bp) added into the droplet in (a), and 12.5 µM Broccoli RNA aptamer and 20 µM DFHBI-1T added into the droplet in (b). For (a) and (b), images are obtained over analysis of seven independent trials with relative humidity ranging from 55% to 65%. (c) The transcription mixture and 50 nM BroccoliT DNA template solution

were added into the ATPS mixture with the volume ratio of 1:1:10. A 5 μL droplet of the mixture was pipetted on the glass substrate for evaporation and imaging. (d) The final mixture was prepared by adding 1 μL of ribozyme substrate (10 μM) and 1 μL of ribozyme RNA transcription product into 38 μL ATPS solution. Then a 5 μL droplet of the final mixture was pipetted onto the glass substrate. For (c) and (d), images are obtained over analysis of three independent trials with relative humidity ranging from 55% to 65%. The scale bar is 500 μm.

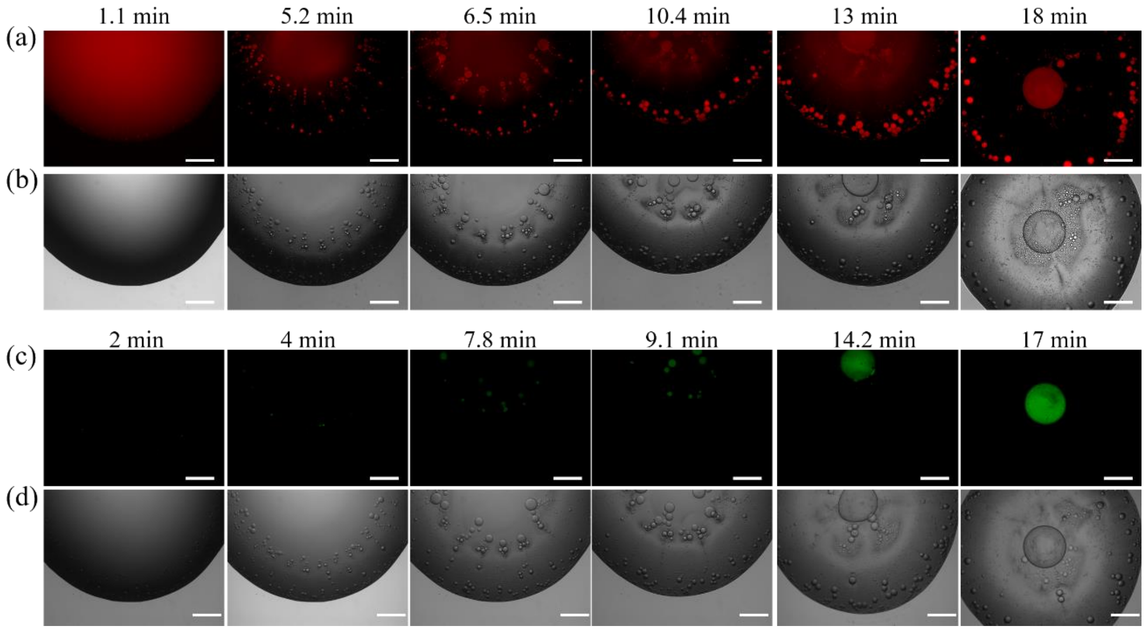

Supplementary Figure 10. Image sequence of BroccoliT RNA aptamer transcription inside the evaporating ATPS droplet. The ATPS mixture composed of 9 *wt%* PEG and 3 *wt%* dextran was used, with the addition of 2 µM CY-5 labeled BroccoliT DNA template. The transcription mixture containing 400 µM DFHBI-1T was added into the ATPS mixture with the volume ratio of 1:3. (a) and (b) show the localization of CY-5 labeled BroccoliT DNA template (2 µM) inside the droplet. Upon *in vitro* transcription of Broccoli DNA template, Broccoli RNA aptamers fluorescently activate the DFHBI-1T fluorophore. (c) and (d) show the fluorescence signals from RNA aptamer and DFHBI-1T complexes. Images are obtained over analysis of three independent trials with relative humidity ranging from 55% to 65%. The scale bar is 500 µm.

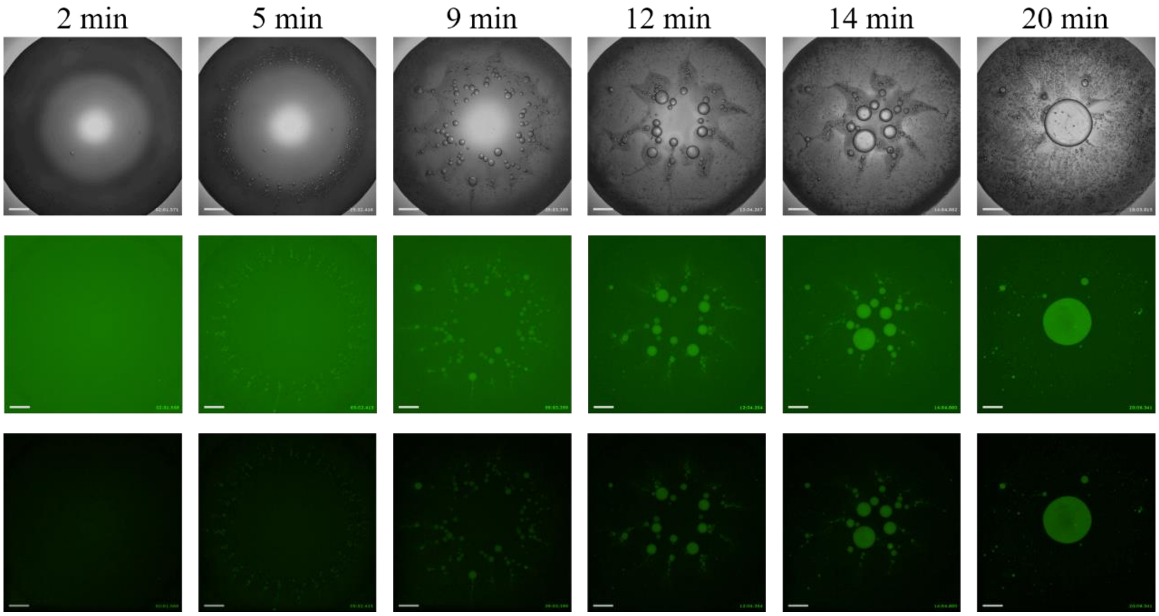

Supplementary Figure 11. Image sequence of hammerhead ribozyme cleavage inside the evaporating ATPS droplet. The upper panel, middle panel and lower panel represent the bright-field, fluorescence and background subtraction image sequence, respectively. Images are obtained over analysis of three independent trials with relative humidity ranging from 55% to 65%. The scale bar is 500 μm.

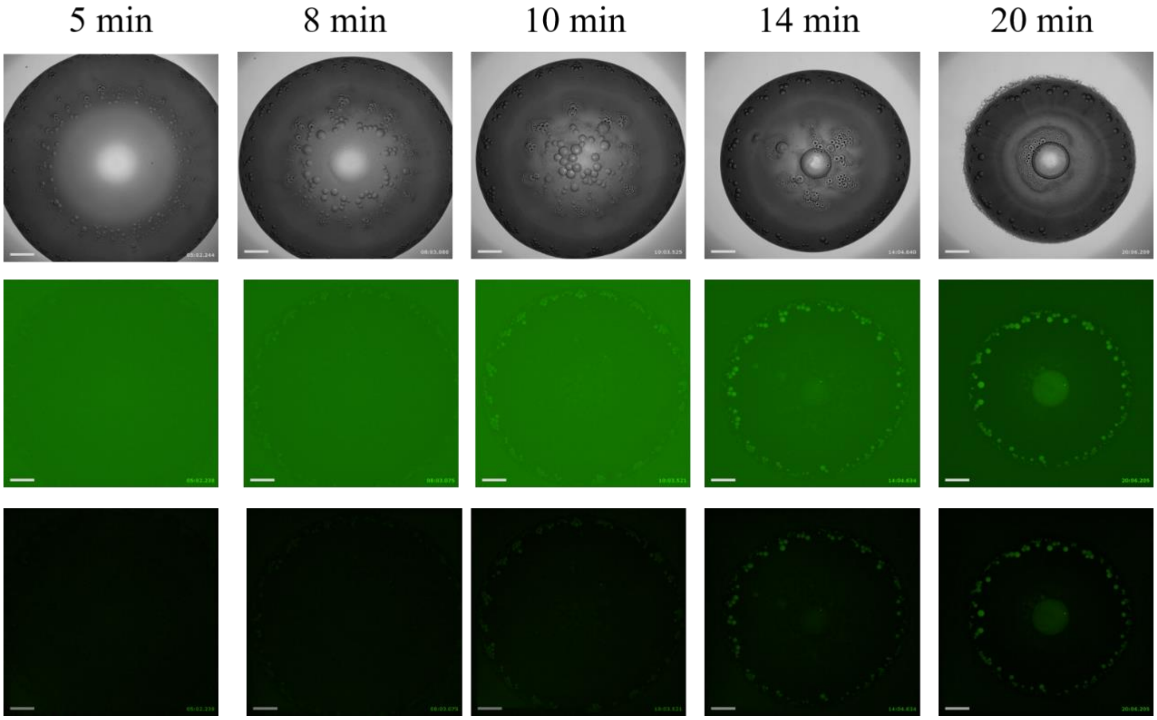

Supplementary Figure 12. Image sequence of control reaction inside the evaporating ATPS droplet, where only fluorophore-labelled substrate without any enzyme strand is present. The upper panel, middle panel and lower panel represent the bright-field, fluorescence and background subtraction image sequence, respectively. Images are obtained over analysis of three independent trials with relative humidity ranging from 55% to 65%. The scale bar is 500 μm.

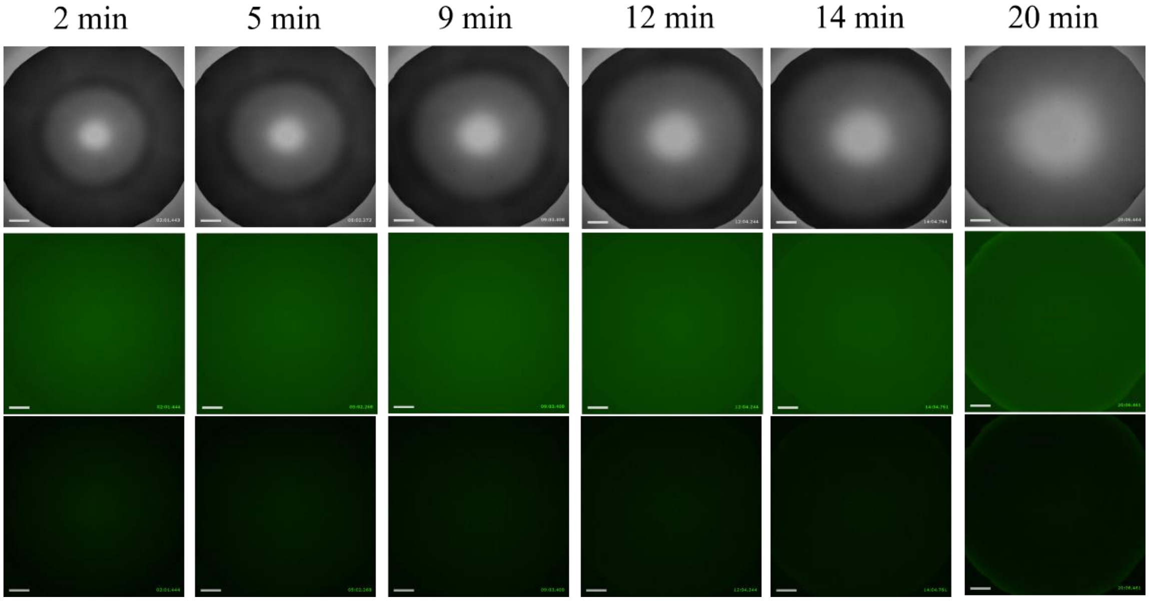

Supplementary Figure 13. Image sequence of control reaction inside the evaporating water droplet, where both fluorophore labelled substrate and enzyme strand have been added. The upper panel, middle panel and lower panel represent the bright-field, fluorescence and background subtraction image sequence, respectively. There is a slight fluorescent “coffee-ring”-like area formed at the edge after evaporation of 20 minutes. The fluorescence level is even lower than that in the ATPS control group (Supplementary Fig. 12). Hence, the cleavage reaction is largely restricted in the sessile droplet of pure water. We attribute the formation of the fluorescent area to the accumulation of fluorophore labelled substrate at the edge due to the outward capillary flow. Images are obtained over analysis of three independent trials with relative humidity ranging from 55% to 65%. The scale bar is 500 μm.

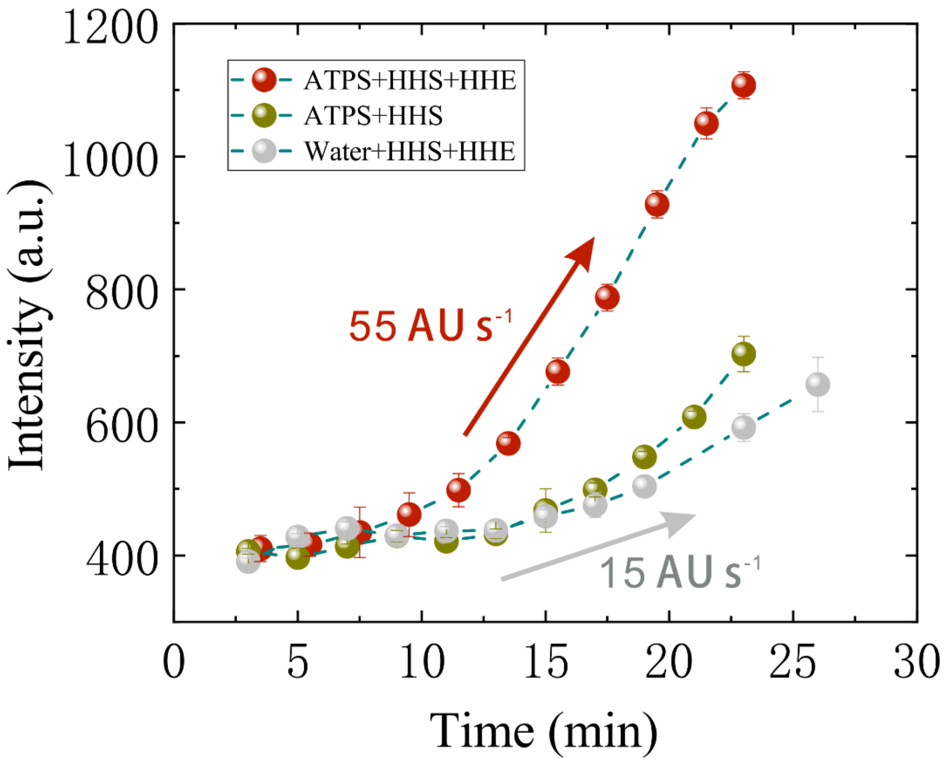

Supplementary Figure 14. Fluorescence intensity (arbitrary unit) inside the sessile droplet as a function of time. Ribozyme activity in the dextran-rich compartments was enhanced more than 3-fold, in comparison to that in the water droplet. Error bars represent S.E.M. from three independent experiments. Source data are provided as a Source Data file.

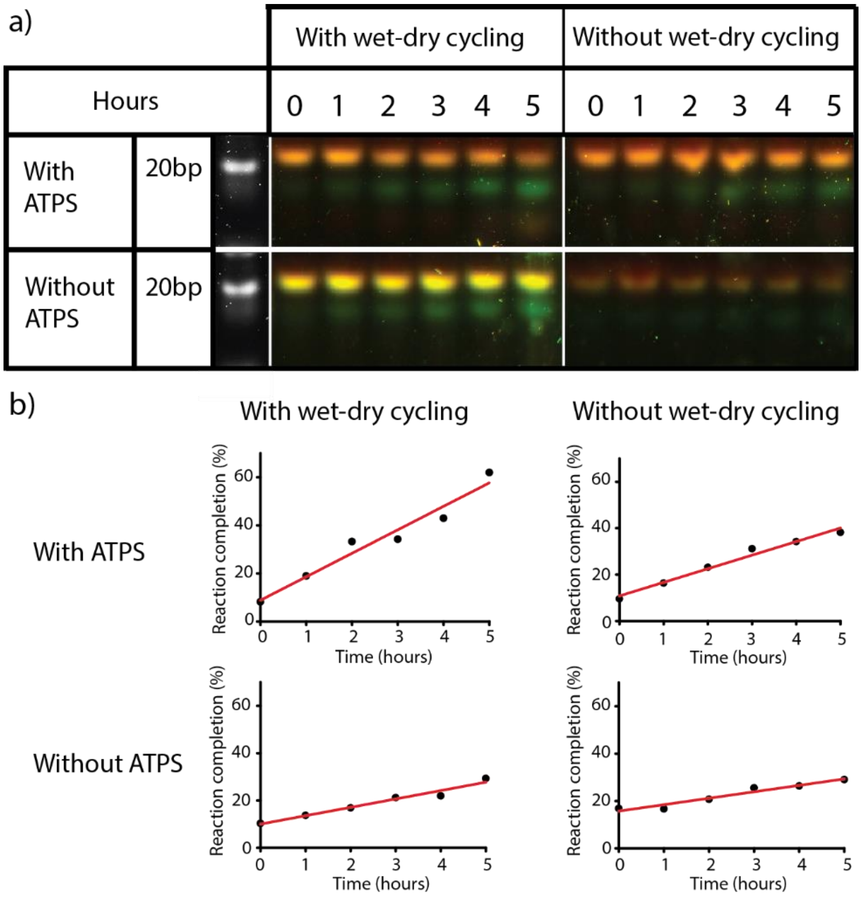

Supplementary Figure 15. Hammerhead ribozyme cleavage activity under wet-dry cycle conditions. Experiments were performed in a time course from left to right of 0, 1, 2, 3, 4 and 5 hours. Gels were processed in parallel. (a) PAGE analysis of ribozyme cleavage reactions. The green signal is FAM and the red signal is TAMRA. The upper orange band is substrate and the lower green and red bands are the cleavage products. (b) Analysis of reaction rate from gel band intensity. The reaction completion was calculated by dividing the product band intensity by the sum of the product and substrate band intensities at ex490/em525. The reaction rate was increased over 3 times for the ATPS and wet-dry cycling condition when compared to the rate without ATPS, without wet-dry cycling condition. Source data are provided as a Source Data file.

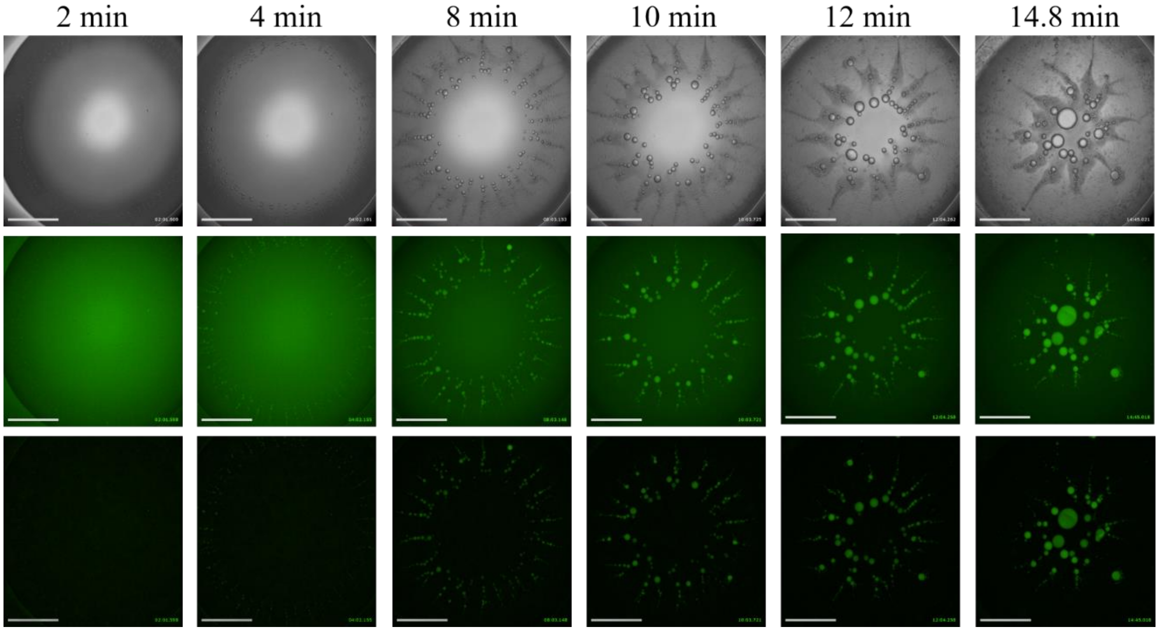

Supplementary Figure 16. Image sequence of X-motif ribozyme cleavage inside the evaporating ATPS droplet. The upper panel, middle panel and lower panel represent the bright-field, fluorescence and background subtraction image sequence, respectively. Images are obtained over analysis of three independent trials with relative humidity ranging from 55% to 65%. The scale bar is 1 mm.

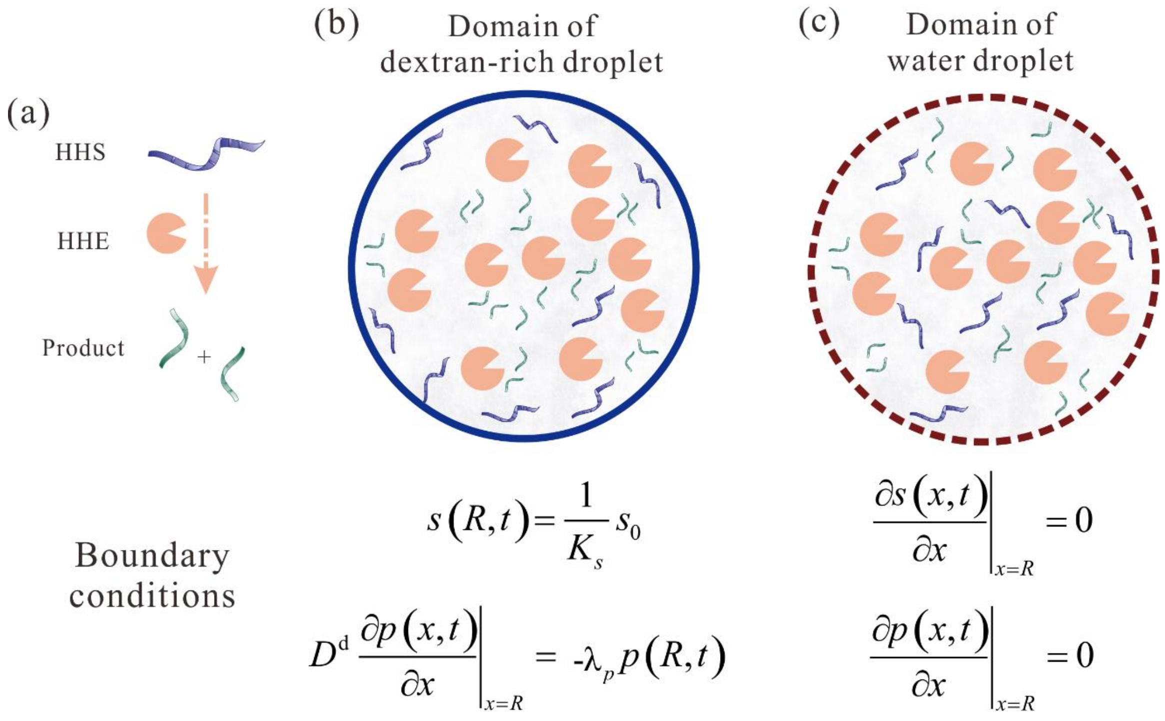

Supplementary Figure 17. Reaction kinetics of the ribozyme cleavage with different boundary conditions. (a) Schematic of the hammerhead substrate (HHS) cleaved into two fragments (Product) by the hammerhead ribozyme (HHE). (b) For the cleavage reaction inside a dextran-rich droplet with a radius R, at the domain boundary, the concentration of the substrate, $s(x,t)$, is assumed to be constant due to the partitioning effects of the dextran-rich compartment, $s(x,t)|_{x=R} = s_0^{\mathrm{d}}$, where s_0^{d} is the concentration of substrate at the boundary of the dextran-rich droplet. This means that the consumption of substrates here caused by the cleavage reaction can be instantaneously compensated by partitioning substrate RNA from surrounding PEG-rich phase. For the concentration of the product, $p(x,t)$, due to the shorter length and weak partitioning strength of short product RNA, it has a specified flux at the boundary, described by $D^{\mathrm{d}} \frac{\partial p(x,t)}{\partial x}\big|_{x=R} = -\lambda_p p(R,t)$, where D^{d} is the diffusion coefficient of the product RNA inside the dextran-rich droplet, and λ_p is the permeability of dextran-rich compartments to the product RNA. (c) For the cleavage reaction inside a water droplet with a radius R, For boundary conditions, owing to the lack of partitioning and compartmentalization effects, $s(x,t)$ and $p(x,t)$ satisfy the same no-flux boundary condition at the edge of the domain. Thus, we have $\frac{\partial s(x,t)}{\partial x}\bigg|_{x=R} = 0$ and $\frac{\partial p(x,t)}{\partial x}\bigg|_{x=R} = 0$.

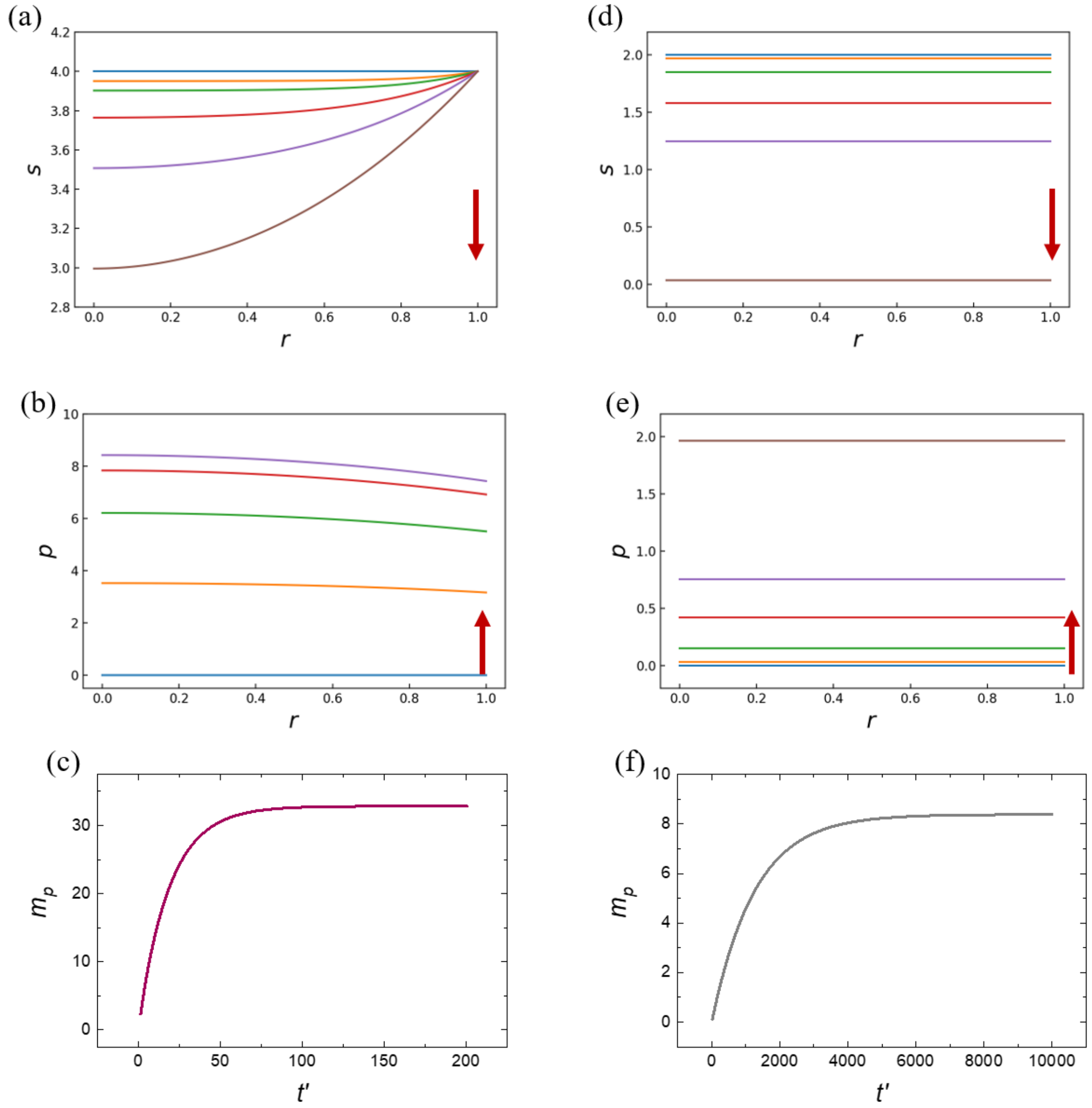

Supplementary Figure 18. Results of the reaction-diffusion models. (a-c) Results of the model for reactions inside dextran-rich compartments, solving from Supplementary Equation 18 and Equation 19: (a) Distribution of ribozyme substrate inside the compartments, with different lines corresponding to results at dimensionless time of $t' =$ 0, 10, 25, 50 and 600, respectively. (b) Distribution of products inside the compartments at the same time point with (a); The red arrow denotes the direction of reaction with time increasing. (c) Productivity of the reaction product as a function of time. (d-f) Results of the model for reactions inside the water compartments, solving from Supplementary Equation 20 and Equation 21: (d) Distribution of ribozyme substrate inside the compartments. with different lines corresponding to results at dimensionless time of $t' =$ 0, 20, 100, 300, 600 and 5000, respectively. (e) Distribution of products inside the compartments at the same time point with (d); The red arrow denotes the direction of reaction with time increasing. (f) Productivity of the reaction product as a function of time. Source data are provided as a Source Data file.

Supplementary Table 1. Parameters in the calculation domain. A spanwise of $0.05R_0$ from the edge of the droplet is selected. c_0 is the initial polymer concentration in the domain. V_0 and M_0 are the volume of the domain and the mass of solution inside the domain, respectively. dM_0/dt is the rate of mass change due to evaporation. c' is the polymer concentration after 5 seconds of water evaporation. c'' is the polymer concentration at the PEG-rich region (formed via the phase separation of the mixture with polymer concentration of c') after the next 5 seconds of water evaporation. The detailed calculation processes are provided in **Supplementary Method 1** below.

$\boldsymbol{c_0}$	$\boldsymbol{V_0}$	$\boldsymbol{M_0}$	$\boldsymbol{dM_0/dt}$	$\boldsymbol{c'}$ **(after** $\boldsymbol{\Delta t = 5}$ **s)**	$\boldsymbol{c''}$ **(after** $\boldsymbol{\Delta t = 10}$ **s)**
PEG:<br>5 wt%<br><br>Dextran:<br>10 wt%	5.48 nL	5.72 μg	-0.194 μg s^{-1}	PEG:<br>6.02 wt%<br><br>Dextran:<br>12.04 wt%	PEG:<br>11.88 wt%<br><br>Dextran:<br>5.93 wt%
PEG:<br>9 wt%<br><br>Dextran:<br>4 wt%		5.63 μg		PEG:<br>10.88 wt%<br><br>Dextran:<br>4.84 wt%	PEG:<br>14.14 wt%<br><br>Dextran:<br>4.06 wt%

Supplementary Table 2. Sequences of ribozymes, BroccoliT DNA template, Cy-5 DNA as well as primers used in this work. All sequences were purchased from Integrated DNA Technology (IDT).

Name	Sequence
Hammerhead Ribozyme DNA template	5'-TTAGGCGCGTTTCGTCCTATTTGGGACTCATCAGCTGGATCCTATAGTGAGTCGTATTATATTGAACAAGCTC-3'
Hammerhead Substrate	/56-TAMN/rCrGrUrGrCrGrUrCrCrUrGrGrArU/36-FAM/
X-Motif Ribozyme DNA template	5'-GTCCTGTCGCTTGGCTCTCTTCCTTGGCTCTCCCTATAGTGAGTCGTATTATATTGAACAAGCTCGTAT-3'
X-Motif Substrate	/5ATTO488N/rArGrUrCrCrUrGrArUrGrGrUrUrArCrUrCrCrArArU/3BHQ_1/
BroccoliT DNA template	ATACGAGCTTGTTCAATATAATACGACTCACTATAGGAAGACGTAGCAAGGCCCGGATAGCTCAGTCGGTAGAGCAGCGGAGACGGTCGGGTCCAGATATTCGTATCTGTCGAGTAGAGTGTGGGCTCCGCGGGTCCAGGGTTCAAGTCCCTGTTCGGGCGCCATGATAGTAAGAGCAATC
Forward primer	ATACGAGCTTGTTCAATA
Cy5 - forward primer	Cy5 - ATACGAGCTTGTTCAATA
Reverse primer	GATTGCTCTTACTATCA
Cy5- DNA	Cy5-AGCAGCACAGAGGTCAGATG

Supplementary Note 1. The dynamics near the three-phase contact line

As shown in Supplementary Fig. 1 (c) and (d), at the beginning of the evaporation, phase separation occurs at three-phase contact line of the droplet, owing to the higher evaporation here, resulting in the formation of sub-microsized structures. For the droplet in regime 1, there are lobe-shaped dextran-rich phase formed near the rim, while dispersed microsized droplets are developed for the droplet in regime 2. These phase-separation-induced structures move towards the centre of the sessile droplet by Marangoni flow, with a phase separation front (PSF) that distinguishes the single-phase region and phase-separated region. For both regimes, the nucleation of dextran-rich droplets near the three-phase contact line is continuously triggered once the polymer concentration surpasses the binodal curve. These nucleated tiny droplets move towards the droplet center, leading to further "thickening" of the dextran-rich phase in regime 1 and the growth of dextran-rich droplets in regime 2. These results show that the higher evaporation rate near the three-phase contact line is the major driven force to trigger phase separation and control the pattern evolution at early times, though the latter is also sensitive to droplet initial compositions. Similar dynamics has been previously studied during the nucleation of oil droplets near the three-phase contact line of an evaporating "Ouzo" droplet [4].

Supplementary Note 2. Size evolution of the phase-separated droplets

The size of phase-separated compartments follows the scaling law of $R_s \sim t$ at the early evaporation stage (t $\leq$100 s). This is consistent with the Siggia's coarsening mechanism, where the hydrodynamic flow is the main transport process.[5] A transition from $R_s \sim t$ to $R_s \sim t^2$ at around 100 s is observed, mainly due to the evaporation-driven decrease of sessile droplet height, resulting in a change in the shape of phase-separated droplets from spherical to cylindrical, and a corresponding increase in the droplet radius.

Supplementary Method 1. Evaporation flux calculation

For a sessile droplet with a shape of a spherical cap, the droplet height h is a function of droplet contact angle θ and droplet radius R, and can be written as

$$h(r,t)=\sqrt{\frac{R^2}{\sin^2\theta}-r^2}-R\cot\theta \tag{1}$$

The droplet evaporation rate can therefore be calculated as

$$\frac{dM}{dt}=-\int_0^R J(r)\sqrt{1+(\partial_r h)^2}\,2\pi dr \tag{2}$$

where $J(r)$ is evaporation flux that is modeled by the vapor diffusion equation $\nabla^2 n=0$. Here the boundary conditions of the diffusion process are set as: $n=n_\infty$ far away from the droplet and $n=n_s$ at the liquid-air interface. Here n_s and n_∞ are the saturated vapor concentration and ambient vapor concertation (decided by humidity $n_\infty=\mathrm{RH}\cdot n_s$, where RH is the relative humidity), respectively. Then the evaporation flux can be written as $J(r)=-D\nabla n$, where D is vapor diffusion coefficient. As a result, the droplet evaporation rate has the analytical solution of

$$\frac{dM}{dt}=-\pi RD(n_s-n_\infty)\Big[\frac{\sin\theta}{1+\cos\theta}+4\int_0^\infty\frac{1+\cosh 2\theta\tau}{\sinh 2\pi\tau}\tanh[\left(\pi-\theta\right)\tau]d\tau\Big] \tag{3}$$

where τ is the integral variable. In our experiments, a sessile droplet of 0.5 µL pipetted on the glass slide has an initial radius of $R_0\approx 1$ mm and the initial contact angle of $\theta\approx 40°$. Substituting $n_s=17.3$ g m^{-3}, and $\mathrm{RH}\approx 60\%$ into the equation, we have $dM/dt\approx 0.9$ µg s^{-1}. This result can be further confirmed based on the evaporation time of a 0.5 µL droplet (with water mass of about 425 µg), which is estimated to be about 400 seconds, with an average evaporation rate of $1.06\,(\pm 0.03)$ µg s^{-1}, consistent with the calculated value from the model. Supplementary Fig. 5 shows the calculated local droplet evaporation rate as a function of R_i based on this model, where the local evaporation rate has a magnitude of

$$\frac{dM_i}{dt}=-\int_0^{R_i} J(r)\sqrt{1+(\partial_r h)^2}\,2\pi dr \tag{4}$$

This suggests that the local evaporation rate along droplet surface is highly non-uniform.

Here we choose a calculation domain with a spanwise of $0.05R_0$ from the edge of the droplet. The volume of the calculation domain, the initial mass of solutions in the calculation domain, and the mass change rate due to evaporation are, respectively,

$$V_0=\int_{0.95R}^{R} h\left(r.t\right)2\pi r dr \tag{5}$$

$$M_0=\rho V_0 \tag{6}$$

$$\frac{dM_0}{dt}=-\int_{0.95R}^{R} J(r)\sqrt{1+(\partial_r h)^2}\,2\pi dr \tag{7}$$

As a result, the polymer concentration after the evaporation time of Δt is

$$c' = M_0 c_0 / \left(M_0 + \frac{dM_0}{dt} \cdot \Delta t \right) \tag{8}$$

where c_0 is the initial polymer concentration. The calculation results for droplets in the two different experiments are listed in **Supplementary Table 1**.

Supplementary Method 2. Marangoni stress calculation

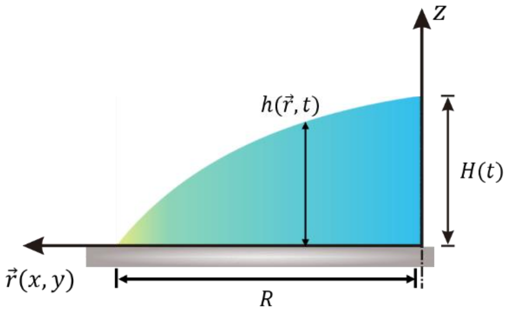

In our model system, the aspect ratio of droplet is $H/R \approx 0.18$. The lubrication approximation can be applied to calculate the flow velocity $\vec{u}$ and volume flux $\vec{Q}$. Here $\vec{Q}$ is defined as

$$\vec{Q}(\vec{r},t) = \int_0^{h(\vec{r},t)} \vec{u}(\vec{r},z,t)dz \tag{9}$$

With the lubrication approximation, $\vec{u}$ and $\vec{Q}$ can be obtained using the following equations:

$$\vec{u}(\vec{r},z,t) = \frac{1}{\mu}\frac{\partial p}{\partial r}\left(\frac{1}{2}z^2 - h(\vec{r},t)z\right) + \frac{z}{\mu}\frac{\partial \gamma}{\partial r} \tag{10}$$

$$\vec{Q}(\vec{r},t) = \frac{h^3}{3\mu}\nabla(\gamma\nabla^2 h) + \frac{h^2}{2\mu}\nabla\gamma \tag{11}$$

where μ is the viscosity, γ is the surface tension, and p is the capillary pressure ($p = -\gamma\nabla^2\tilde{h}$), respectively.

The first term of $\vec{Q}(\vec{r},t)$ indicates the capillary flow induced by the gradient of Laplace pressure. The second term denotes the Marangoni flow driven by the Marangoni effects. Therefore, for sufficiently small $\Delta\gamma$ or $\Delta\gamma/\gamma$, the magnitude of capillary flow and Marangoni flow can be estimated as

$$Q_{\mathrm{Ca}} \sim \frac{\gamma H^4}{\mu R^3},\ Q_{\mathrm{Ma}} \sim \frac{\Delta\gamma}{\mu}\frac{H^2}{R} \tag{12}$$

The ratio of these two terms is estimated as

$$\frac{Q_{\mathrm{Ma}}}{Q_{\mathrm{Ca}}} \sim \frac{R^2\Delta\gamma}{H^2\gamma} \tag{13}$$

Substituting the experimental parameters: $H \approx 170$ μm, $R \approx 0.96$ mm, $\gamma \approx 63.5$ mN m^{-1}, and then $\Delta\gamma$ that satisfies $Q_{\mathrm{Ma}}/Q_{\mathrm{Ca}} = 1$ is calculated to be $\Delta\gamma \approx 2$ mN m^{-1}.

This indicates that with small H/R, a small Marangoni force is sufficient to suppress the outward capillary flow. In our model system, a surface tension difference of 2 mN m^{-1} along the droplet surface can be easily achieved due to non-uniform evaporation rate. For example, for droplets composed of 5 wt% PEG and 10 wt% dextran, the initial surface tension is measured to be 63.5 mN m^{-1}. After a few seconds of evaporation, the composition

near the droplet edge is expected to be close to that in the top PEG-rich phase of the mixture containing 30 wt% PEG and 1 wt% dextran, with a surface tension of about 60 mN m^{-1}. This results in a surface tension difference of 3.5 mN m^{-1}.

Supplementary Method 3. Reaction-diffusion kinetics of ribozyme cleavage

1) Reaction-diffusion kinetics inside the domain of dextran-rich phase

For ease of notation, the superscript "d" and "w" denote the values of variables inside the domain of dextran-rich droplet and water droplet, respectively. The reaction-diffusion equations for kinetics inside the domain of dextran-rich compartment are written as

$$\frac{\partial s(x,t)}{\partial t} = D^{\mathrm{d}}\nabla^2 s(x,t) - \frac{k_{\mathrm{cat}} e_0^{\mathrm{d}} s(x,t)}{K_{\mathrm{M}} + s(x,t)} \tag{14}$$

$$\frac{\partial p(x,t)}{\partial t} = D^{\mathrm{d}}\nabla^2 p(x,t) + \frac{k_{\mathrm{cat}} e_0^{\mathrm{d}} s(x,t)}{K_{\mathrm{M}} + s(x,t)} \tag{15}$$

where D^{d} is the RNA diffusion constant inside the domain of dextran-rich compartment, and e_0^{d} is the concentration of ribozyme. k_{cat} and K_{M} denote catalytic rate and Michaelis constant respectively, as described in Equation 3 and Equation 4 in the main text. For both $s(x,t)$ and $p(x,t)$, due to the spherical symmetry of the domain, they satisfy the same no-flux boundary condition of $\left.\frac{\partial s(x,t)}{\partial x}\right|_{x=0} = 0$ and $\left.\frac{\partial p(x,t)}{\partial x}\right|_{x=0} = 0$ at the centre of the domain. As shown in the Supplementary Fig. 17(b), at the domain boundary, $s(x,t)$ is assumed to be constant due to the partitioning effects of the dextran-rich compartment, $s(x,t)|_{x=R} = s_0^{\mathrm{d}}$, which means that the consumption of substrates here caused by the cleavage reaction can be instantaneously compensated by partitioning substrate RNA from surrounding PEG-rich phase. For $p(x,t)$, due to the short length induced weak partitioning strength of the product RNA, it has a specified flux at the boundary, described by $D^{\mathrm{d}}\left.\frac{\partial p(x,t)}{\partial x}\right|_{x=R} = -\lambda_p p(R,t)$, where λ_p is the permeability of dextran-rich compartments to the product RNA. With the $p(x,t)$ solving from the model, the productivity of ribozyme cleavage reaction is given by integrating $p(x,t)$ over the domain $M_p(t) = \int_0^R 4\pi x^2 \cdot p(x,t)dx$.

2) Reaction-diffusion kinetics inside the domain of water

Similarly, the reaction-diffusion model for ribozyme cleavage reaction in water droplet is derived as

$$\frac{\partial s(x,t)}{\partial t} = D^{\mathrm{w}}\nabla^2 s(x,t) - \frac{k_{\mathrm{cat}} e_0^{\mathrm{w}} s(x,t)}{K_{\mathrm{M}} + s(x,t)} \tag{16}$$

$$\frac{\partial p(x,t)}{\partial t} = D^{\mathrm{w}} \nabla^2 p(x,t) + \frac{k_{\mathrm{cat}} e_0^{\mathrm{w}} s(x,t)}{K_{\mathrm{M}} + s(x,t)} \tag{17}$$

where D^{w} is the RNA diffusion constant inside the water droplet, and e_0^{w} is the concentration of ribozyme. For boundary conditions, owing to the lack of partitioning effects, $s(x,t)$ and $p(x,t)$ satisfy the same no-flux boundary condition both at the centre and the edge of the domain, as shown in the Supplementary Fig. 17(c) above. Thus, we have $\left.\frac{\partial s(x,t)}{\partial x}\right|_{x=0} = 0$, $\left.\frac{\partial s(x,t)}{\partial x}\right|_{x=R} = 0$, $\left.\frac{\partial p(x,t)}{\partial x}\right|_{x=0} = 0$ and $\left.\frac{\partial p(x,t)}{\partial x}\right|_{x=R} = 0$.

3) Derivation of models with the dimensionless form

To compare the reaction-diffusion kinetics in the dextran-rich droplet and water droplet, the reaction-diffusion equations need to be recast into the non-dimensional forms. By defining a characteristic length scale, droplet radius R_0, a time scale $t_0 = R_0^2 / D^{\mathrm{w}}$, and a characteristic RNA concentration e_0 ($e_0 = e_0^{\mathrm{w}}$), Supplementary Equation 14 and Equation 15 can be rewritten into the dimensionless forms

$$\frac{\partial s(r,t')}{\partial t'} = \frac{D^{\mathrm{d}}}{D^{\mathrm{w}}} \nabla^2 s(r,t') - \frac{\alpha s(r,t')}{\beta + s(r,t')} \cdot \frac{1}{K_e} \tag{18}$$

$$\frac{\partial p(r,t')}{\partial t'} = \frac{D^{\mathrm{d}}}{D^{\mathrm{w}}} \nabla^2 p(r,t') + \frac{\alpha s(r,t')}{\beta + s(r,t')} \cdot \frac{1}{K_e} \tag{19}$$

where $r = x / R_0$, $t' = t / t_0$, $\alpha = \frac{k_{\mathrm{cat}} R_0^2}{D^{\mathrm{w}}}$, $\beta = \frac{K_{\mathrm{M}}}{e_0}$ and K_e is the partitioning coefficient of the ribozyme in the PEG/dextran system ($K = [\mathrm{RNA}]_{\text{PEG-rich}} / [\mathrm{RNA}]_{\text{Dextran-rich}}$), with the boundary conditions in dimensionless form

$$\begin{cases} \left.\frac{\partial s(r,t')}{\partial r}\right|_{r=0} = 0 \\ s(r,t')\big|_{r=1} = (1/K_s) \cdot s_0 / e_0 \end{cases} \quad \begin{cases} \left.\frac{\partial p(r,t')}{\partial r}\right|_{r=0} = 0 \\ \left.\frac{\partial p(r,t')}{\partial r}\right|_{r=1} = -\frac{\lambda_p R}{D^{\mathrm{d}}} p(1,t') \end{cases} \quad \begin{cases} p(r,0) = 0 \\ s(1,0) = (1/K_s) s_0 / e_0 \end{cases}$$

where $s_0 = s_0^{\mathrm{w}}$ and K_s is the partitioning coefficient of the substrate in the PEG/dextran system.

Similarly, Supplementary Equation 16 and Equation 17 can be non-dimensionlized as

$$\frac{\partial s(r,t')}{\partial t'} = \nabla^2 s(r,t') - \frac{\alpha s(r,t')}{\beta + s(r,t')} \tag{20}$$

$$\frac{\partial p(r,t')}{\partial t} = \nabla^2 p(r,t') + \frac{\alpha s(r,t')}{\beta + s(r,t')} \tag{21}$$

and the boundary conditions become

$$\begin{cases} \left.\dfrac{\partial s(r,t')}{\partial r}\right|_{r=0} = 0 \\ \left.\dfrac{\partial s(r,t')}{\partial r}\right|_{r=1} = 0 \end{cases} \quad \begin{cases} \left.\dfrac{\partial p(r,t')}{\partial r}\right|_{r=0} = 0 \\ \left.\dfrac{\partial p(r,t')}{\partial r}\right|_{r=1} = 0 \end{cases} \quad \begin{cases} p(r,0) = 0 \\ s(1,0) = s_0 / e_0 \end{cases}$$

Supplementary Equation 18 to Equation 21 can be solved numerically with a finite-element method. Finally, the non-dimensional productivity of ribozyme cleavage reaction is given by $m_p(t') = \int_0^1 4\pi r^2 \cdot p(r,t')\,dr$.

Through the comparison of Supplementary Equation 18 to Equation 19, and Supplementary Equation 20 to Equation 21, we find that there are two major factors that distinct the reaction-diffusion kinetics inside dextran-rich droplets and water droplets. One is the partitioning of ribozyme and substrate into the dextran-rich compartments, with the appearance of K_e in Supplementary Equation 18 and Equation 19 and K_s in their boundary conditions. The other difference lies in the expression of boundary conditions, with the constant substrate concentration and permeability of product RNA at the domain boundary of dextran-rich compartment.

These differences lead to distinct results of the reaction-diffusion kinetic models in the two types of domains. As shown in Supplementary Fig. 18, over time, the distribution of substrate and product in dextran-rich compartment is significantly different from that inside the water droplet (Supplementary Fig. 18(a-b) and Supplementary Fig. 18(d-e)), which is consistent with the RNA partitioning properties of dextran-rich compartments. Also, the reaction rate inside the dextran-rich compartment is more than 50 times faster than that of the water droplet, with the final reaction productivity in the dextran-rich domain about 4

times higher than that in water droplets (Supplementary Fig. 18(c) and Supplementary Fig. 18(f)). The comparison is also given in Figure 5(e) in the main text. With the reaction-diffusion kinetic models, it can be concluded that the dextran-rich compartments formed through segregative phase separation have significant advantages over water droplets in both accelerating and promoting ribozyme cleavage, which arise from a spatial localization and enrichment of the reactants induced by phase separation.

4) *Physical constants*

The physical constants and coefficients in the model are obtained experimentally ($s_0 = 0.25\ \mu M\ and\ e_0 = 0.125\ \mu M$) or from literatures[6-8] (parameters listed below).

$$K_e = 0.0062;\ K_s = 0.5$$

$$K_\mathrm{M} = 6.0\ \mu\mathrm{M};\ k_\mathrm{cat} = 2.35\ \mathrm{min}^{-1}$$

$$D^\mathrm{d} = 20\ \mu\mathrm{m}^2\mathrm{s}^{-1};\ D^\mathrm{w} = 100\ \mu\mathrm{m}^2\mathrm{s}^{-1};\ \lambda_p = 0.1\ \mu\mathrm{m\ s}^{-1}$$

Supplementary References

1. Song, B. & Springer, J. Determination of Interfacial Tension from the Profile of a Pendant Drop Using Computer-Aided Image Processing: 1. Theoretical. *Journal of Colloid and Interface Science* **184**, 64-76 (1996).
2. Song, B. & Springer, J. Determination of Interfacial Tension from the Profile of a Pendant Drop Using Computer-Aided Image Processing: 2. Experimental. *Journal of Colloid and Interface Science* **184**, 77-91 (1996).
3. Balsara, N.P., Lin, C. & Hammouda, B. Early Stages of Nucleation and Growth in a Polymer Blend. *Physical Review Letters* **77**, 3847-3850 (1996).
4. Tan, H*., et al.* Evaporation-triggered microdroplet nucleation and the four life phases of an evaporating Ouzo drop. *Proceedings of the National Academy of Sciences of the United States of America* **113**, 8642-8647 (2016).
5. Siggia, E.D. Late stages of spinodal decomposition in binary mixtures. *Physical Review A* **20**, 595-605 (1979).
6. Dewey, D.C., Strulson, C.A., Cacace, D.N., Bevilacqua, P.C. & Keating, C.D. Bioreactor droplets from liposome-stabilized all-aqueous emulsions. *Nat Commun* **5**, 4670 (2014).
7. Strulson, C.A., Molden, R.C., Keating, C.D. & Bevilacqua, P.C. RNA catalysis through compartmentalization. *Nature Chemistry* **4**, 941-946 (2012).
8. Dix, J.A. & Verkman, A.S. Crowding effects on diffusion in solutions and cells. *Annu Rev Biophys* **37**, 247-263 (2008).
