# Supplementary material for: Non-associative phase separation in an evaporating droplet as a model for prebiotic compartmentalization
Source: Nat Commun. 2021 May 27;12:3194. doi: 10.1038/s41467-021-23410-7 (PMC8160217; doi:10.1038/s41467-021-23410-7)
Supplement: Supplementary file 3 — Description of Additional Supplementary Files [file 41467_2021_23410_MOESM3_ESM.pdf]

Description of Additional Supplementary Files

File name: Supplementary Movie 1
Description: Polymer self-organization and compartmentalization inside an evaporating ATPS droplet in Regime 1. For fluorescence imaging, 0.01 mg/mL fluorescein isothiocyanate–dextran (FITC-dextran, 4 kg/mol, Sigma-Aldrich) and 1 mg/mL Rhodamine B (>=95% (HPLC), Sigma-Aldrich) were added into the mixture.

File name: Supplementary Movie 2
Description: Polymer self-organization and compartmentalization inside an evaporating ATPS droplet in Regime 2. For fluorescence imaging, 0.01 mg/mL fluorescein isothiocyanate–dextran (FITC-dextran, 4 kg/mol, Sigma-Aldrich) and 1 mg/mL Rhodamine B (>=95% (HPLC), Sigma-Aldrich) were added into the mixture.

File name: Supplementary Movie 3
Description: DNA localization and compartmentalization inside an evaporating ATPS droplet. DNA was labelled with Cy5 dye (red fluorescence) and dextran-rich droplets are labelled with FITC-dextran.

File name: Supplementary Movie 4
Description: RNA (aptamer) localization and compartmentalization inside an evaporating ATPS droplet. Broccoli RNA aptamer and DFHBI fluorophore were introduced into the mixture for fluorescence imaging.

File name: Supplementary Movie 5
Description: In-vitro transcription (IVT) inside an evaporating ATPS droplet. BroccoliT DNA template and DFHBI fluorophore were introduced into the mixture. Broccoli RNA aptamers transcribe from their DNA templates and complex with DFHBI, emitting green fluorescence.

File name: Supplementary Movie 6
Description: Hammerhead ribozyme cleavage inside an evaporating ATPS droplet.
